# Older Age Threshold for Oxaliplatin Benefit in Stage II to III Colorectal Cancer

**DOI:** 10.1001/jamanetworkopen.2025.25660

**Published:** 2025-08-06

**Authors:** Jun Woo Bong, Hwamin Lee, Seogsong Jeong, Sanghee Kang

**Affiliations:** 1 Department of Surgery, Korea University Guro Hospital, Korea University College of Medicine, Seoul, Republic of Korea; 2 Department of Biomedical Informatics, Korea University College of Medicine, Seoul, Republic of Korea

## Abstract

**Importance:**

Colorectal cancer is a leading cause of cancer mortality, with increasing incidence in older adults. Oxaliplatin-based adjuvant chemotherapy is standard for stage II to III colorectal cancer, but its benefit in older patients remains unclear.

**Objective:**

To investigate whether there is an optimal age threshold for a survival benefit of adding oxaliplatin to fluoropyrimidine-based adjuvant chemotherapy in older patients with stage II to III colorectal cancer.

**Design, Setting, and Participants:**

This population-based, retrospective cohort study using data from the Korea Health Insurance Review and Assessment Service included patients who underwent curative resection for stage II to III colorectal cancer and received adjuvant chemotherapy between January 2014 and December 2016. Participants were followed up until date of death or April 30, 2024, whichever occurred earlier.

**Exposures:**

Oxaliplatin-based adjuvant chemotherapy compared with fluoropyrimidine-only chemotherapy.

**Main Outcomes and Measures:**

The primary outcome was overall survival, analyzed using Cox proportional hazards regression and propensity score matching. Age thresholds from 60 to 80 years were systematically assessed to identify the optimal cutoff for oxaliplatin benefit. The association between chemotherapy discontinuation and oxaliplatin use was evaluated using multivariate regression analysis.

**Results:**

Among a total of 53 147 patients, 8561 (mean [SD] age, 63.2 [11.2] years; 5084 [59.4%] male) were included in this study (stage II: 2913 [34.0%]; stage III: 5648 [65.9%]). Oxaliplatin was not associated with improved survival among patients with stage II disease across all age thresholds (adjusted hazard ratios [AHRs] ranged from 0.71 [95% CI, 0.34-1.50] to 1.09 [95% CI, 0.73-1.64]). In patients with stage III disease, oxaliplatin was associated with significantly improved survival up to age 70 years (AHR, 0.59; 95% CI, 0.46-0.77; *P* < .001), with a 5-year overall survival rate of 84.8% in the oxaliplatin group and 78.1% in the nonoxaliplatin group (*P* = .003). In patients older than 70 years, oxaliplatin was not associated with survival (AHR, 0.85; 95% CI, 0.67-1.07; *P* = .18). Multivariate regression analysis showed that oxaliplatin use was significantly associated with chemotherapy discontinuation in patients older than 70 years with stage III disease (adjusted odds ratio [AOR], 1.55; 95% CI, 1.19-2.03; *P* = .001), whereas no such association was observed in patients aged 70 years or younger (AOR, 1.22; 95% CI, 0.93-1.62; *P* = .16).

**Conclusions and Relevance:**

In this population-based cohort study, oxaliplatin addition was associated with significantly improved survival among patients with stage III colorectal cancer aged 70 years or younger but not in those older than 70 years. There was no association with improved survival among patients with stage II disease regardless of age. Moreover, in patients older than 70 years with stage III disease, oxaliplatin use was significantly associated with chemotherapy discontinuation.

## Key Points

**Question:**

Is there an optimal age threshold for a survival benefit of adding oxaliplatin to fluoropyrimidine-based adjuvant chemotherapy among older patients with stage II to III colorectal cancer?

**Findings:**

In this cohort study of 8561 Korean patients, oxaliplatin was associated with improved overall survival among patients aged 70 years or younger with stage III disease but not among those older than 70 years or those with stage II disease.

**Meaning:**

The findings suggest that oxaliplatin may benefit patients aged 70 years or younger with stage III colorectal cancer, while its use in patients aged older than 70 years and those with stage II disease warrants reconsideration.

## Teaser

This cohort study investigates whether the addition of oxaliplatin to adjuvant chemotherapy for stage II to III colorectal cancer is associated with improved survival at age thresholds from 60 to 80 years and whether there is an optimal age threshold for survival benefit.

## Introduction

Advances in treatment and screening have led to declining age-adjusted mortality from colorectal cancer, while the disease burden remains high in the growing older population.^1^ Although surgical resection with adjuvant chemotherapy is the primary treatment for nonmetastatic colorectal cancer, the role of adjuvant chemotherapy in older patients remains controversial, particularly regarding the addition of oxaliplatin to fluoropyrimidine-based regimens.

The benefits of oxaliplatin-based combination chemotherapy in older patients have been the subject of ongoing debate, with conflicting evidence. The MOSAIC trial subgroup analysis did not show a substantial survival benefit for patients aged 65 years or older,^2^ and the analysis of the ACCENT database revealed no improvement in disease-free or overall survival (OS) with oxaliplatin administration in older patients.^3^ However, a large retrospective study showed improved OS associated with additional oxaliplatin administration in patients older than 70 years (hazard ratio [HR], 0.6; 95% CI, 0.5-0.9).^4^ Moreover, a pooled analysis of randomized trials showed a reduced but substantial benefit in older patients, including those with comorbidities.^5^ Treatment decision-making is complicated by age-related factors, with different treatment responses and toxic effect profiles observed in older patients than in other cohorts.^6^ In particular, oxaliplatin-induced neurotoxic effects are more severe and persistent in older patients.^7^ This study used data from a national registry to evaluate the current practice of adding oxaliplatin to fluoropyrimidine-based adjuvant chemotherapy regimens for stage II to III colorectal cancer for older patients and to assess whether there is an optimal age threshold for survival benefit.

## Methods

### Study Population

This population-based, retrospective cohort study used data from the Korea Health Insurance Review and Assessment Service (HIRA) National Quality Assessment (NQA) program. The HIRA was established to review health care benefit claims and perform adequate evaluations of health care services under the National Health Insurance system.^8,9^ The HIRA-NQA establishes objective targets by considering the characteristics of each evaluation criterion and applying national policy goals and clinical practice guidelines. The HIRA-NQA collects hospital-based clinical data from Statistics Korea, including demographic characteristics; medical treatments, such as surgery and chemotherapy; surgical characteristics; and the date of death. This study was approved by the institutional review board of Korea University Guro Hospital, and the requirement for informed consent was waived because this retrospective study used anonymized data provided by the HIRA and did not involve any direct contact with patients or access to identifiable personal information. The study was conducted according to the Strengthening the Reporting of Observational Studies in Epidemiology (STROBE) reporting guideline for cohort studies.

The HIRA-NQA database includes patients who underwent curative radical resection for stage II to III colorectal cancer by postoperative pathologic confirmation and received adjuvant chemotherapy between January 2014 and December 2016. Patients were excluded if they (1) received chemotherapy other than the nonoxaliplatin or oxaliplatin-combined treatments listed in the following section (ie, participation in clinical trials of new chemotherapy); (2) received neoadjuvant treatments, including preoperative chemoradiotherapy for rectal cancer; (3) received adjuvant chemotherapy 2 months or more after surgery rather than within 2 months after surgery; and/or (4) had missing information. We categorized patients by disease stage (II or III) and compared survival outcomes between nonoxaliplatin and oxaliplatin use.

### Adjuvant Chemotherapy

This study examined the outcomes associated with adjuvant chemotherapy regimens for stage II and III colorectal cancer, including nonoxaliplatin treatments (fluorouracil plus leucovorin, capecitabine) and oxaliplatin-combined treatment (capecitabine plus oxaliplatin [CAPOX], fluorouracil plus leucovorin plus oxaliplatin [FOLFOX], and modified FOLFOX [described in eTable 2 in Supplement 1]). Eligible patients received 1 of these adjuvant chemotherapy regimens following curative radical resection. Patients who received other chemotherapy regimens were excluded. We evaluated the proportion of patients who entirely discontinued chemotherapy before completing planned cycles and defined discontinuation as a ratio of actual cycles to planned cycles of less than 1.

### Key Variables

For the adjusted analyses, the following variables were considered: age (years), sex (female, male), body mass index (BMI; calculated as weight in kilograms divided by height in meters squared and categorized as underweight [<18.5], normal weight [18.5-22.9], and overweight [≥23.0]), emergency operation for colorectal cancer (yes, no), Charlson Comorbidity Index (CCI), American Society of Anesthesiologists (ASA) Physical Status Classification, tumor histology (adenocarcinoma, mucinous adenocarcinoma, or signet ring cell carcinoma), number of harvested lymph nodes (<12, ≥12), capecitabine-based regimen (yes, no), and chemotherapy discontinuation (yes, no).

### Statistical Analysis

All participants were followed up from the date of surgery until the date of death or April 30, 2024, whichever occurred earlier. Categorical and continuous variables were presented as number (percentage) and mean (SD), respectively. Cox proportional hazards regression analysis was used to evaluate the association between adjuvant chemotherapy and OS in older patients with colorectal cancer. To determine the age thresholds of older people who could attain survival benefits from oxaliplatin, we evaluated adjusted HRs (AHRs) and 95% CIs for age thresholds ranging from 60 to 80 years.

One crude and 2 adjusted models were developed: a minimally adjusted model accounting for age and sex and a fully adjusted model incorporating adjustments for age, sex, BMI, emergency operation for colorectal cancer, CCI, ASA classification, tumor histology, number of harvested lymph nodes, capecitabine-based regimen, and chemotherapy discontinuation. Mortality was calculated as deaths per 100 person-years.

As a sensitivity analysis, patients who died within 6 months or 1 year from the start of follow-up were excluded to minimize potential uncontrolled bias. Then, 1:1 propensity score matching (PSM) was carried out with a caliper value of 0.1 and with age, sex, BMI, emergency operation for colorectal cancer, CCI, ASA classification, tumor histology, number of harvested lymph nodes, capecitabine-based regimen, and chemotherapy discontinuation as covariates. The adequacy of PSM was evaluated by examining the standardized mean difference, with a cutoff value less than 0.1 indicating acceptable matching quality.

Kaplan-Meier estimation was used to assess OS probabilities among the nonoxaliplatin and oxaliplatin adjuvant chemotherapy groups following PSM. The log-rank test was used to determine statistical significance. Stratified analyses were conducted by categorizing patients based on sex, BMI, emergency operation for colorectal cancer, CCI, ASA classification, tumor histology, number of harvested lymph nodes, capecitabine-based regimen, and chemotherapy discontinuation to examine the interactions between these variables and adjuvant chemotherapy in association with OS. Additionally, multivariate logistic regression analysis was performed to evaluate adjusted odds ratios (AORs) to determine the association between oxaliplatin use and chemotherapy discontinuation, adjusting for age, sex, BMI, emergency operation for colorectal cancer, CCI, ASA classification, tumor histology, number of harvested lymph nodes, and capecitabine-based regimen.

Differences with 2-sided *P* < .05 were considered statistically significant. All data collection, mining, and analyses were performed using R, version 3.4.5 (R Project for Statistical Computing).

## Results

### Patient Characteristics

**Figure 1.**
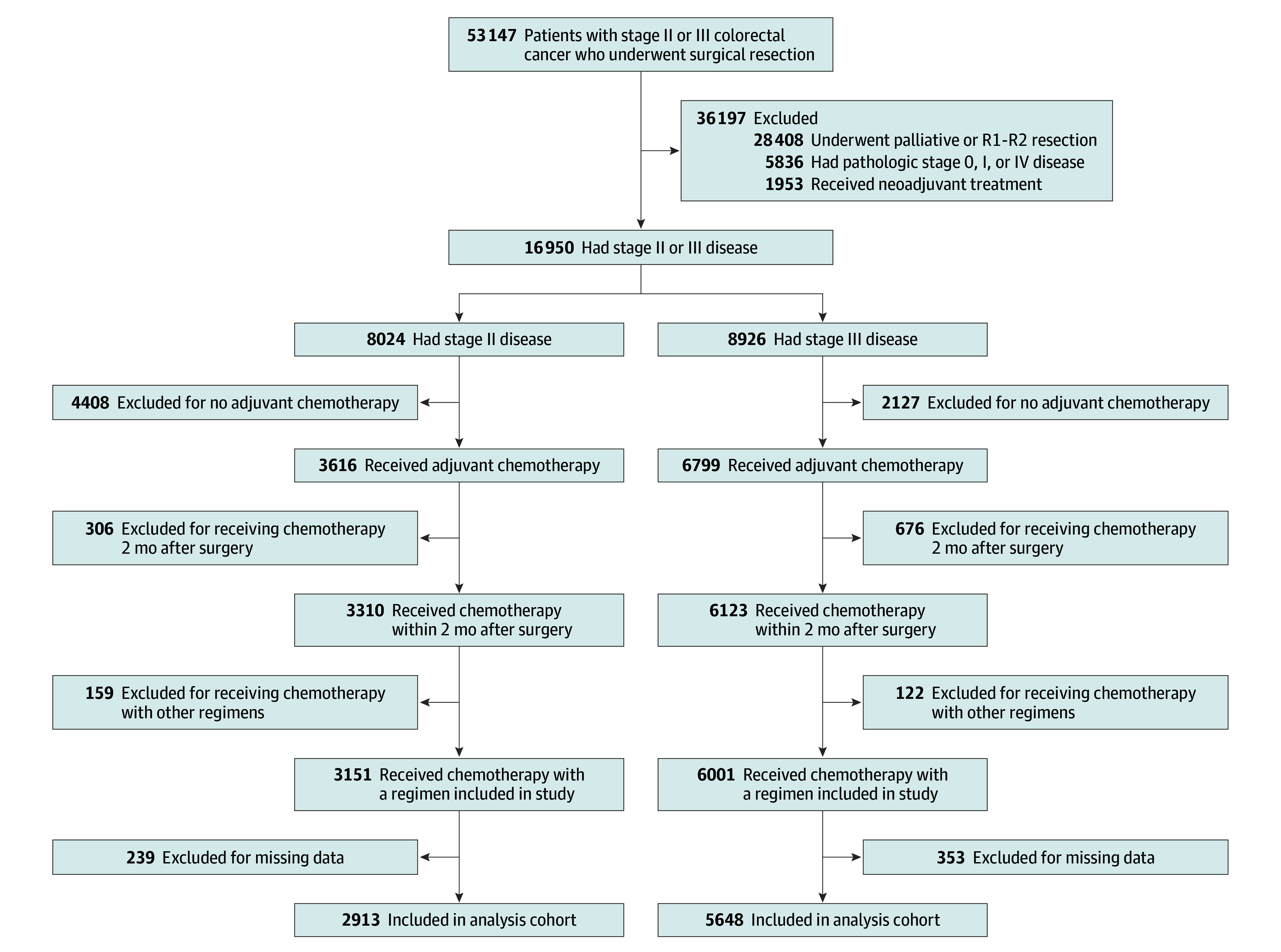
Flow Diagram for the Inclusion of the Study Population Includes patients who underwent curative radical resection for colorectal cancer between 2014 and 2016 from the Korea Health Insurance Review and Assessment Service’s National Quality Assessment program database.

The clinical data of 53 147 patients were collected, and 44 586 patients were excluded according to the exclusion criteria, leaving 8561 patients in the analysis cohort (mean [SD] age, 63.2 [11.2] years; 3477 [40.6%] female and 5084 [59.4%] male) (Figure 1). eTables 1 and 2 in Supplement 1 show the descriptive characteristics of the study patients according to pathologic stage and the detailed chemotherapy regimens used in each group, respectively. A total of 2913 (34.0%) and 5648 (65.9%) patients had stage II or III disease, respectively. The proportions of females, patients with obesity, patients using oxaliplatin-based regimens, and patients who discontinued chemotherapy were higher in the group with stage III disease than in the group with stage II disease. Additionally, oral capecitabine regimens were used more frequently in patients with stage III disease (1334 [23.6%]) than in those with stage II disease (631 [21.6%]) (*P* = .04). Of 3915 individuals aged 70 years or younger, 352 (8.9%) received nonoxaliplatin treatment compared with 662 of 1717 (38.6%) older than 70 years (eTables 3 and 4 in Supplement 1).

### Age Thresholds for Oxaliplatin-Based Chemotherapy Based on OS According to Pathologic Stage

**Figure 2.**
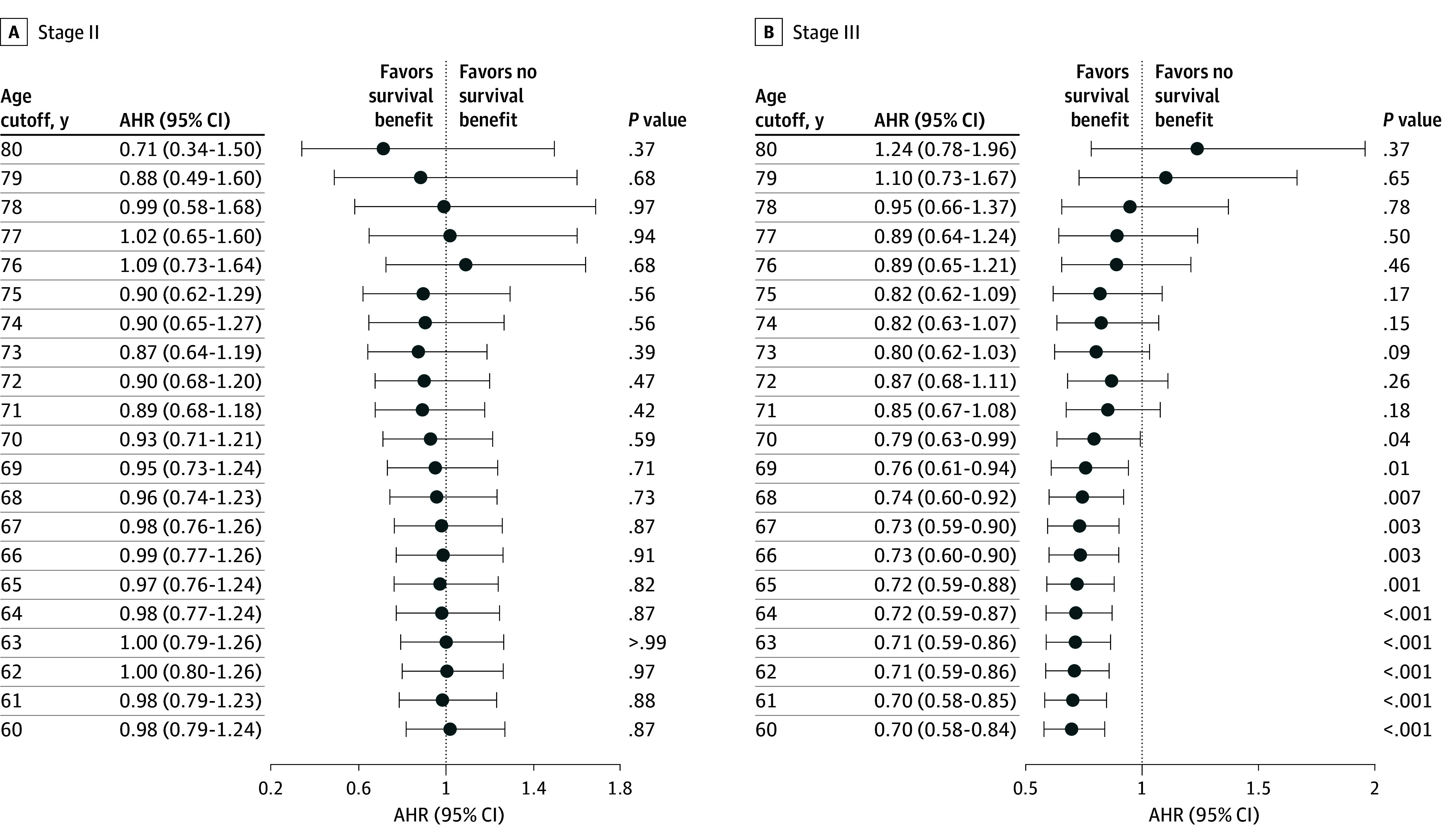
Adjusted Hazard Ratios (AHRs) for Overall Survival by Use of Oxaliplatin-Combined vs Nonoxaliplatin Adjuvant Chemotherapy, Stratified by Cancer Stage and Age at Diagnosis Analysis was performed using Cox proportional hazards regression models adjusted for age, sex, body mass index, emergency operation for colorectal cancer, Charlson Comorbidity Index, American Society of Anesthesiologists classification, tumor histology, lymph node count, chemotherapy regimen, and treatment.

For patients with stage II disease, we could not identify a significant age threshold ranging from 60 to 80 years in which oxaliplatin was associated with improved survival compared with nonoxaliplatin treatment (AHRs ranged from 0.71 [95% CI, 0.34-1.50] to 1.09 [95% CI, 0.73-1.64]) (Figure 2A). In contrast, for patients with stage III disease, our analysis revealed 70 years as the critical age threshold. Oxaliplatin was associated with improved survival among patients up to age 70 years (AHR at the age threshold of 70 years, 0.79; 95% CI, 0.63-0.99; *P* = .04), but there was no association when examining age thresholds above 71 years (Figure 2B).

### Association of Oxaliplatin With OS in Patients With Stage III Disease After Stratification by Age Threshold Before and After PSM

In the fully adjusted model of patients aged 70 years or younger with stage III disease, the AHR for the association of oxaliplatin use with OS was 0.59 (95% CI, 0.46-0.77; *P* < .001) (Table 1); however, in patients older than 70 years, oxaliplatin use was not associated with improved OS in any model (eg, fully adjusted model: AHR, 0.85; 95% CI, 0.67-1.07; *P* = .18). eTables 3 and 4 in Supplement 1 show the PSM results for each age group. After PSM, the AHR for the association of oxaliplatin use with OS in the fully adjusted model of patients aged 70 years or younger was 0.60 (95% CI, 0.41-0.89; *P* = .01). In patients older than 70 years, oxaliplatin was not associated with improved survival in any model. The sensitivity analysis, which excluded events within specified periods, showed similar results (eTable 5 in Supplement 1). In the stratified analysis, oxaliplatin therapy was associated with a lower risk of mortality compared with nonoxaliplatin regimens in patients aged 70 years or younger across several subgroups. However, no consistent survival benefit was observed among patients older than 70 years, and there were no significant interactions between treatment effect and subgroup characteristics (eTable 6 in Supplement 1).

**Table 1.** Association Between Oxaliplatin Use and Overall Mortality Among Patients With Pathologic Stage III Colorectal Cancer According to Age Cutoff Before and After PSM^a^

Outcome	Before PSM			After PSM		
	Nonoxaliplatin	Oxaliplatin	*P* value	Nonoxaliplatin	Oxaliplatin	*P* value
**Age ≤70 y**						
Patients, No.	352	3563	NA	346	346	NA
Events, No.	104	806	NA	102	72	NA
Person-years	2680	27 947	NA	2638	2724	NA
Mortality rate per 100 person-years, % (95% CI)	3.8 (3.1-4.7)	2.8 (2.6-3.0)	NA	3.8 (3.1-4.6)	2.6 (2.1-3.4)	NA
Crude HR (95% CI)	1 [Reference]	0.73 (0.60-0.90)	.003	1 [Reference]	0.68 (0.50-0.92)	.01
Minimally adjusted HR (95% CI)^b^	1 [Reference]	0.79 (0.64-0.97)	.03	1 [Reference]	0.67 (0.49-0.90)	.009
Fully adjusted HR (95% CI)^c^	1 [Reference]	0.59 (0.46-0.77)	<.001	1 [Reference]	0.60 (0.41-0.89)	.01
**Age >70 y**						
Patients, No.	662	1055	NA	300	300	NA
Events, No.	310	469	NA	147	130	NA
Person-years	4370	7070	NA	1975	1992	NA
Mortality rate per 100 person-years, % (95% CI)	7.0 (6.3-7.9)	6.6 (6.0-7.2)	NA	7.4 (6.4-8.8)	6.6 (6.2-8.7)	NA
Crude HR (95% CI)	1 [Reference]	0.93 (0.81-1.08)	.36	1 [Reference]	0.88 (0.70-1.16)	.29
Minimally adjusted HR (95% CI)^b^	1 [Reference]	1.14 (0.98-1.33)	.08	1 [Reference]	0.91 (0.72-1.15)	.45
Fully adjusted HR (95% CI)^c^	1 [Reference]	0.85 (0.67-1.07)	.18	1 [Reference]	0.87 (0.64-1.17)	.38

Abbreviations: HR, hazard ratio; NA, not applicable; PSM, propensity score matching.

^a^ Among 3915 patients aged 70 years or younger, 3563 (91.0%) received oxaliplatin and 352 (9.0%), nonoxaliplatin. Among 1717 patients older than 70 years, 1055 (61.4%) received oxaliplatin and 662 (38.6%), nonoxaliplatin. HRs were evaluated using Cox proportional hazards regression.

^b^ Adjusted for age and sex.

^c^ Adjusted for age, sex, body mass index, emergency operation for colorectal cancer, Charlson Comorbidity Index, American Society of Anesthesiologists classification, tumor histology, number of harvested lymph nodes, capecitabine-based regimen, and chemotherapy discontinuation.

### Kaplan-Meier Curves for OS in Patients With Stage III Disease in Each Age Group Before and After PSM

**Figure 3.**
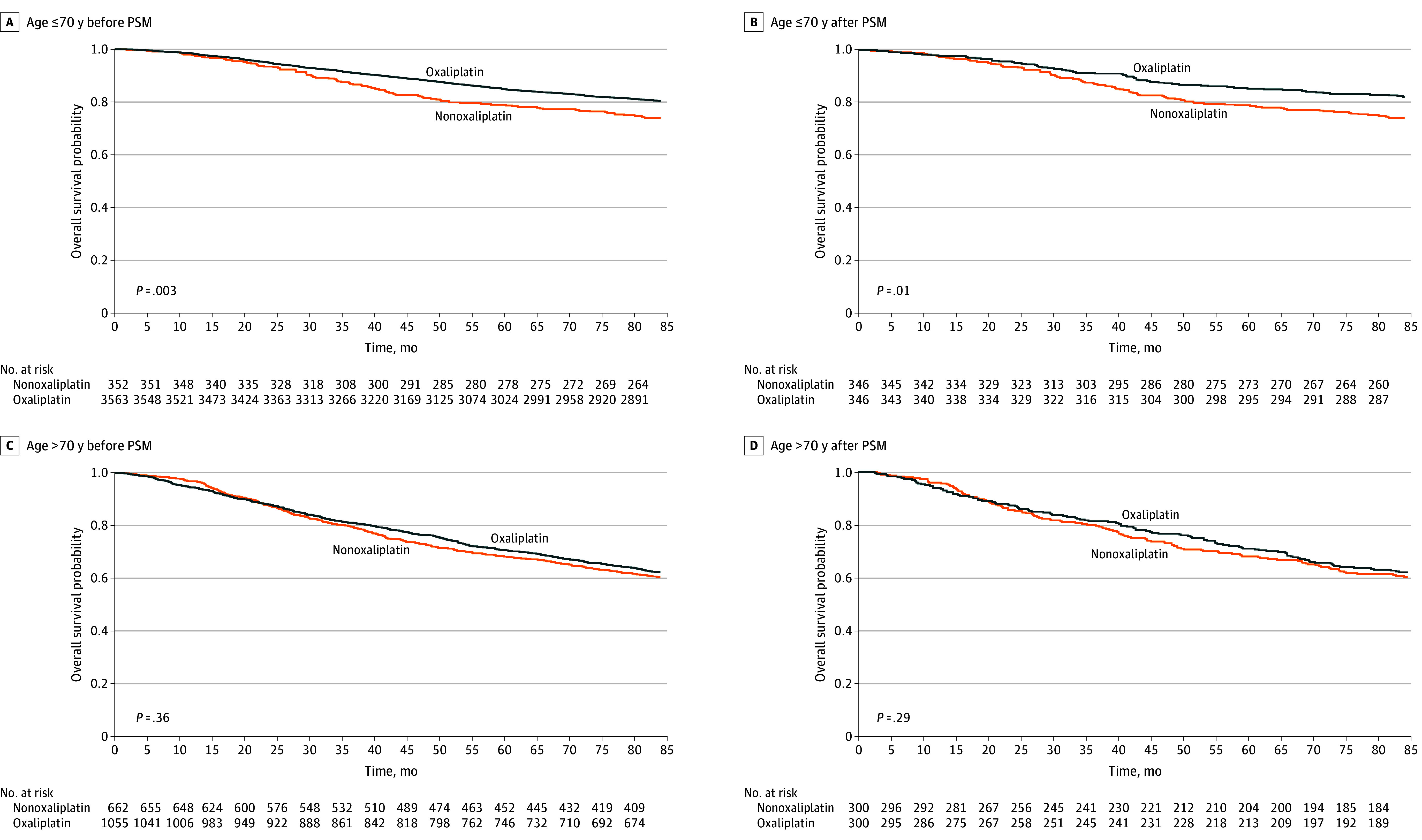
Kaplan-Meier Survival Curves by Age and Oxaliplatin Use in Patients With Stage III Disease Kaplan-Meier estimates comparing overall survival between oxaliplatin and nonoxaliplatin adjuvant chemotherapy before and after propensity score matching (PSM). *P* values were derived from log-rank tests.

As shown in Figure 3A, for patients with stage III disease aged 70 years or younger before PSM, 5-year OS in the nonoxaliplatin and oxaliplatin groups was 78.1% and 84.8%, respectively (*P* = .003). After PSM, 5-year OS in the oxaliplatin group was higher than that in the nonoxaliplatin group (85.0% vs 78.9%; *P* = .01) (Figure 3B). As shown in Figure 3C, 5-year OS among patients older than 70 years in the nonoxaliplatin and oxaliplatin groups before PSM was 68.3% and 70.6%, respectively (*P* = .36). After PSM, 5-year OS in the nonoxaliplatin and oxaliplatin groups did not show a significant difference (68.0% vs 71.0%; *P* = .29).

### Association Between Oxaliplatin and Chemotherapy Discontinuation in Patients With Stage III Disease After Stratification by Age

The rate of chemotherapy discontinuation among patients older than 70 years with stage III disease (642 of 1717 [37.4%]) was higher than that among patients aged 70 years or younger (935 of 3915 [23.9%]) (*P* < .001). The eFigure in Supplement 1 illustrates the increasing rates of chemotherapy discontinuation as age increased from 60 to 80 years. Additionally, in patients older than 70 years, the rate of chemotherapy discontinuation in the oxaliplatin group was significantly higher than that in the nonoxaliplatin group (421 of 1055 [39.9%] vs 221 of 662 [33.4%]; *P* = .008) (eTable 3 in Supplement 1). In patients aged 70 years or younger, the discontinuation rate in the oxaliplatin group was not significantly different from that in the nonoxaliplatin group (862 of 3563 [24.2%] vs 73 of 352 [20.7%]; *P* = .17) (eTable 4 in Supplement 1). Multivariate regression analysis showed that for patients aged 70 years or younger, oxaliplatin use was not significantly associated with chemotherapy discontinuation (AOR, 1.22; 95% CI, 0.93-1.62; *P* = .16) (Table 2). However, in patients older than 70 years, oxaliplatin was significantly associated with chemotherapy discontinuation (AOR, 1.55; 95% CI, 1.19-2.03; *P* = .001). Chemotherapy discontinuation was significantly associated with worse OS across both age groups. For patients aged 70 years or younger, the AHR was 1.79 (95% CI, 1.56-2.06; *P* < .001), and for those older than 70 years, the AHR was 1.54 (95% CI, 1.33-1.79; *P* < .001) (eTable 7 in Supplement 1). Furthermore, in patients aged 70 years or younger, discontinuation of oxaliplatin while continuing fluoropyrimidine was associated with worse OS when the oxaliplatin dose decreased below 80% (AHR, 1.72; 95% CI, 1.12-2.62; *P* = .01) (eTable 8 in Supplement 1). However, this association was not observed in patients older than 70 years.

**Table 2.** Multivariate Analysis of the Association Between Oxaliplatin Use and Chemotherapy Discontinuation in Patients With Stage III Colorectal Cancer According to Age Cutoff

Variable	Age ≤70 y		Age >70 y	
	AOR (95% CI)^a^	*P* value	AOR (95% CI)^a^	*P* value
Oxaliplatin use				
No	1 [Reference]	NA	1 [Reference]	NA
Yes	1.22 (0.93-1.62)	.16	1.55 (1.19-2.03)	.001
Sex				
Female	1 [Reference]	NA	1 [Reference]	NA
Male	1.05 (0.90-1.22)	.52	0.82 (0.67-1.00)	.06
BMI				
Normal	1 [Reference]	NA	1 [Reference]	NA
Underweight	0.89 (0.61-1.27)	.53	1.40 (0.92-2.14)	.11
Overweight	0.85 (0.73-0.99)	.049	0.93 (0.75-1.15)	.51
Emergency operation for colorectal cancer				
Yes	1 [Reference]	NA	1 [Reference]	NA
No	1.09 (0.75-1.62)	.66	0.32 (0.18-0.54)	<.001
Charlson Comorbidity Index				
0	1 [Reference]	NA	1 [Reference]	NA
1-2	0.95 (0.80-1.13)	.59	1.27 (0.96-1.68)	.09
3-4	1.15 (0.90-1.45)	.24	1.53 (1.11-2.11)	.008
≥5	0.93 (0.66-1.29)	.70	1.47 (1.00-2.16)	.054
ASA classification				
1	1 [Reference]	NA	1 [Reference]	NA
2	1.08 (0.91-1.29)	.33	0.83 (0.56-1.25)	.38
≥3	1.47 (1.10-1.94)	.006	1.17 (0.76-1.81)	.47
Histologic type				
Adenocarcinoma	1 [Reference]	NA	1 [Reference]	NA
Others	0.97 (0.70-1.31)	.74	1.54 (0.93-2.54)	.09
Harvested lymph nodes, No.				
<12	1 [Reference]	NA	1 [Reference]	NA
≥12	1.19 (0.75-1.31)	.43	1.70 (0.99-2.89)	.055
Capecitabine-based regimen				
No	1 [Reference]	NA	1 [Reference]	NA
Yes	0.93 (0.76-1.14)	.53	1.24 (0.95-1.63)	.11

Abbreviations: AOR, adjusted odds ratio; ASA, American Society of Anesthesiologists; BMI, body mass index (calculated as weight in kilograms divided by height in meters squared); NA, not applicable.

^a^ Evaluated using logistic regression analysis.

## Discussion

This study used national data to analyze the age threshold from 60 through 80 years for benefit from oxaliplatin as a chemotherapy adjuvant compared with a nonoxaliplatin regimen in patients with colorectal cancer. The analysis results showed that oxaliplatin treatment was not associated with improved survival among patients with stage III disease aged older than 70 years or those with stage II disease. However, oxaliplatin treatment was associated with improved OS among patients with stage III colorectal cancer who were aged 70 years or younger. Moreover, among patients with stage III disease, chemotherapy discontinuation was significantly associated with oxaliplatin use in patients older than 70 years, whereas no such association was found in patients aged 70 years or younger.

Oxaliplatin-based regimens have shown significant toxicity, particularly neurotoxicity, making it essential to evaluate their benefits in older patients.^10^ Furthermore, understanding how age thresholds affect treatment outcomes can guide therapeutic decision-making in this vulnerable population.^11^ This study found no association of oxaliplatin use with improved survival in patients older than 70 years. This highlights the importance of refining age thresholds and incorporating geriatric assessments into treatment planning to identify patients who are likely to benefit from aggressive regimens.^12^ Although studies often set this threshold at 65 or 70 years of age, physiologic age, comorbidities, and functional status may better reflect the ability of an individual to tolerate treatment. Studies have suggested that biological age, which includes factors such as frailty and organ function, may be more relevant than chronological age in predicting chemotherapy outcomes.^13^

The lack of an association between oxaliplatin-based regimens and survival in patients with stage II disease may be attributable to several factors. First, the absolute risk reduction of recurrence may be lower in patients with stage II disease than in those with stage III disease, in which lymph node involvement increases the risk of recurrence. Second, patients with stage II disease often have a better baseline prognosis, which potentially reduces the incremental benefits of adding oxaliplatin. Studies, including the SCOT trial,^14^ showed that shorter treatment durations with oxaliplatin regimens had limited efficacy in patients with high-risk stage II colorectal cancer, emphasizing the need to balance efficacy with the toxic effects associated with longer durations of therapy for patients with stage II disease. Additionally, the ACHIEVE-2 trial^15^ revealed that 3-month durations of oxaliplatin-based adjuvant chemotherapy were associated with reduced rates of toxic effects; however, noninferiority of outcomes with shorter regimens compared with longer-duration regimens was not conclusively established in populations with stage II disease, especially for the FOLFOX regimen. Furthermore, a study analyzing reduced-dose administration of oxaliplatin reported no significant differences in survival outcomes when doses were lowered in patients with stage II disease, suggesting that lower recurrence risks in these patients may reduce the need for intensive oxaliplatin-based regimens.^16^ Overall, these findings highlight the complex interplay among the efficacy, toxic effects, and baseline prognosis for patients with stage II disease when considering adjuvant chemotherapy strategies.

How oxaliplatin can be appropriately used in older patients should be considered. The SCOT trial^17^ highlighted that shorter durations of oxaliplatin-based therapy (3 months vs 6 months) could significantly reduce the incidence of grade 2 or greater neurotoxic effects without compromising efficacy in patients with stage III disease, thus offering a safer alternative for older patients. The ACHIEVE-2 trial^15^ further demonstrated that for older patients with stage III disease, treatment with a 3-month regimen of CAPOX provided noninferior outcomes compared with 6-month regimens, with lower rates of toxic effects. These findings underscore the importance of individualized treatment planning to balance efficacy and safety, particularly for older patients with stage III disease.

Investigating therapeutic strategies to maximize the efficacy of chemotherapy and minimize dose modifications in older patients is necessary. Older patients often experience dose delays, reductions, or discontinuations due to adverse effects, such as neurologic and gastrointestinal toxic effects.^18^ Moreover, Table 2 indicates that the use of oxaliplatin was significantly associated with discontinuation of chemotherapy in patients aged 71 to 80 years. eTable 8 in Supplement 1 presents the percentage of patients who discontinued oxaliplatin without discontinuing fluoropyrimidine treatment. In patients aged 70 years or younger, discontinuing oxaliplatin showed no significant correlation with overall survival when the oxaliplatin dose was maintained at 80% or higher. However, we were unable to assess these results in patients aged more than 70 years due to the small numbers in the subgroups.

Individualized strategies are critical for achieving optimal outcomes in this population and for ensuring that efficacy and safety are effectively balanced. The ACCORE study^19^ showed that a reduction in chemotherapy dose intensity in older patients was not associated with survival outcomes, suggesting that tailored dosing strategies may effectively balance toxic effects and adherence to treatment. Additionally, lower relative dose intensities of oxaliplatin in older patients have been associated with reduced frequency of severe adverse effects, such as neutropenia and neuropathy, allowing more patients to complete their prescribed therapy.^20^ A multicenter study reported that older patients with metastatic colorectal cancer who underwent comprehensive geriatric assessments before initiating chemotherapy had better treatment adherence and fewer severe toxic effects owing to regimens tailored based on their frailty levels.^21^

The MOSAIC trial^22^ indicated an improvement in survival after the addition of oxaliplatin to 5-fluorouracil plus leucovorin for stage III colorectal cancer; however, this advantage was not observed in patients aged 70 years or older, who encountered more toxic effects. Findings from the IDEA collaboration suggested that a 3-month regimen of oxaliplatin-based adjuvant chemotherapy could maintain efficacy in low-risk patients with stage III disease while lowering rates of neurotoxic effects.^5^ Subgroup analyses in that pooled study indicated that shorter regimens could be tolerated in patients aged 70 years or older; nonetheless, the survival benefit of oxaliplatin in this age group was still restricted, and adherence was low. Consistent with these findings, our population-based analysis of a nationwide cohort revealed that oxaliplatin administration in patients older than 70 years was associated with a significantly elevated rate of treatment discontinuation and was not associated with improved survival, suggesting a necessity for more tailored treatment strategies in older adults.

### Limitations

This study had certain limitations that warrant consideration. First, the retrospective nature of the analysis introduced selection bias and incomplete data, which could have impacted the generalizability of our findings. As shown in eTables 3 and 4 in Supplement 1, only 352 of all 3915 patients aged 70 years or younger (8.9%) underwent nonoxaliplatin treatment, contrasting with the proportion observed in patients older than 70 years (662 of 1717 [38.6%]). This bias may have influenced the statistical outcomes even after PSM. Furthermore, whereas oxaliplatin may enhance recurrence-free survival,^5^ prognostic data beyond OS were not available in this database. Second, detailed molecular and genetic markers (eg, microsatellite instability and *KRAS*) were not included, limiting the ability to explore individual variability. Third, the study did not evaluate adverse effects, including toxic effects and quality-of-life outcomes, which are particularly relevant for older populations, for whom balancing efficacy and tolerability is crucial.

## Conclusions

In this cohort study in a Korean population, oxaliplatin was associated with improved OS among patients with stage III colorectal cancer aged 70 years or younger but not among patients older than 70 years or patients with stage II disease. Oxaliplatin was also associated with higher chemotherapy discontinuation rates in patients older than age 70 years, suggesting that its role remains limited and careful consideration of individual factors associated with these outcomes is warranted. In patients with stage II disease, the lack of association between oxaliplatin and improved survival highlights the need for refined risk stratification to guide adjuvant therapy decisions. Future research should continue to explore innovative approaches to optimize the treatment of older patients with colorectal cancer to ensure a balance between efficacy and safety.

## Notes

Supplement 1. **eTable 1.** Clinical Characteristics of Patients With Colorectal Cancer According to the Pathological Stage **eTable 2.** Chemotherapy Regimens According to Pathological Stage **eTable 3.** Clinical Characteristics of Patients With Stage III Colon Cancer Aged >70 Years According to Oxaliplatin Use Before and After Adjustment Using Propensity Score Matching **eTable 4.** Clinical Characteristics of Patients With Stage III Colon Cancer ≤70 Years Old According to Oxaliplatin Use Before and After Adjustment Using Propensity Score Matching **eTable 5.** Sensitivity Analyses on the Risk of Overall Mortality in Patients With Stage III Colon Cancer After Excluding Events That Occurred Within Specified Periods After the Propensity Score Matching **eTable 6.** Stratified Analyses of Overall Survival in Patients With Stage III Colon Cancer Receiving Non-Oxaliplatin Vs Oxaliplatin-Combined Adjuvant Chemotherapy After Propensity Score Matching **eTable 7.** Adjusted Hazard Ratio of Chemotherapy Discontinuation for Overall Survival According to Age Cut-Off **eTable 8.** Oxaliplatin Dose and Adjusted Hazard Ratio for Overall Survival in Patient With Oxaliplatin Discontinuation According to Age Cut-Off **eFigure.** Discontinuation Rates According to Age in Patients With Stage III Disease

Supplement 2.
Data Sharing Statement
