# Supplementary material for: Older Age Threshold for Oxaliplatin Benefit in Stage II to III Colorectal Cancer
Source: JAMA Netw Open. 2025 Aug 6;8(8):e2525660. doi: 10.1001/jamanetworkopen.2025.25660 (PMC12329608; doi:10.1001/jamanetworkopen.2025.25660)
Supplement: Supplement 1. — eTable 1. Clinical Characteristics of Patients With Colorectal Cancer According to the Pathological Stage eTable 2. Chemotherapy Regimens According to Pathological Stage eTable 3. Clinical Characteristics of Patients With Stage III Colon Cancer Aged >70 Years According to Oxaliplatin Use Before and After Adjustment Using Propensity Score Matching eTable 4. Clinical Characteristics of Patients With Stage III Colon Cancer ≤70 Years Old According to Oxaliplatin Use Before and After Adjustment Using Propensity Score Matching eTable 5. Sensitivity Analyses on the Risk of Overall Mortality in Patients With Stage III Colon Cancer After Excluding Events That Occurred Within Specified Periods After the Propensity Score Matching eTable 6. Stratified Analyses of Overall Survival in Patients With Stage III Colon Cancer Receiving Non-Oxaliplatin Vs Oxaliplatin-Combined Adjuvant Chemotherapy After Propensity Score Matching eTable 7. Adjusted Hazard Ratio of Chemotherapy Discontinuation for Overall Survival According to Age Cut-Off eTable 8. Oxaliplatin Dose and Adjusted Hazard Ratio for Overall Survival in Patient With Oxaliplatin Discontinuation According to Age Cut-Off eFigure. Discontinuation Rates According to Age in Patients With Stage III Disease [file jamanetwopen-e2525660-s001.pdf]

Supplementary Online Content

Bong JW, Lee H, Jeong S, Kang S. Older age threshold for oxaliplatin benefit in stage II to III colorectal cancer. *JAMA Netw Open*. 2025;8(8):e2525660.
doi:10.1001/jamanetworkopen.2025.25660

eTable 1. Clinical Characteristics of Patients With Colorectal Cancer According to the Pathological Stage

eTable 2. Chemotherapy Regimens According to Pathological Stage

eTable 3. Clinical Characteristics of Patients With Stage III Colon Cancer Aged >70 Years According to Oxaliplatin Use Before and After Adjustment Using Propensity Score Matching

eTable 4. Clinical Characteristics of Patients With Stage III Colon Cancer ≤70 Years Old According to Oxaliplatin Use Before and After Adjustment Using Propensity Score Matching

eTable 5. Sensitivity Analyses on the Risk of Overall Mortality in Patients With Stage III Colon Cancer After Excluding Events That Occurred Within Specified Periods After the Propensity Score Matching

eTable 6. Stratified Analyses of Overall Survival in Patients With Stage III Colon Cancer Receiving Non-Oxaliplatin Versus Oxaliplatin-Combined Adjuvant Chemotherapy After Propensity Score Matching

eTable 7. Adjusted Hazard Ratio of Chemotherapy Discontinuation for Overall Survival According to Age Cut-Off

eTable 8. Oxaliplatin Dose and Adjusted Hazard Ratio for Overall Survival in Patient With Oxaliplatin Discontinuation According to Age Cut-Off

eFigure. Discontinuation Rates According to Age in Patients With Stage III Disease

This supplementary material has been provided by the authors to give readers additional information about their work.

© 2025 Bong JW et al. *JAMA Network Open*.

eTable 1. Clinical characteristics of patients with colorectal cancer according to the pathological stage

Characteristics	Stage II<br>N = 2,913 (%)	Stage III<br>N = 5,648 (%)	P-value
Age, years	63.0 ± 11.0	63.4 ± 11.3	0.11
Sex, male	1,799 (61.8)	3,285 (58.2)	0.001
BMI, kg/m^2			0.03
Normal	1,207 (41.5)	2,193 (38.9)	
Underweight	165 (5.7)	294 (5.2)	
~~Obese~~Overweight	1,537 (52.8)	3,148 (55.9)	
Emergency operation	150 (5.1)	224 (4.0)	0.01
CCI			0.30
0	902 (31.0)	1,727 (30.6)	
1−2	1,340 (46.0)	2,579 (45.7)	
3−4	482 (16.5)	912 (16.1)	
≥5	189 (6.5)	430 (7.6)	
ASA classification			0.28
1	609 (20.9)	1,187 (21.0)	
2	1,876 (64.5)	3,708 (65.7)	
≥3	422 (14.5)	749 (13.3)	
Histological type			0.04
Adenocarcinoma	2,788 (95.7)	5,421 (96.0)	
Mucinous adenocarcinoma	121 (4.2)	203 (3.6)	
Signet-ring cell carcinoma	4 (0.1)	24 (0.4)	
Number of harvested lymph nodes <12	102 (3.5)	158 (2.8)	0.08
Chemotherapy regimen			
Oxaliplatin based	1,332 (45.7)	4,633 (82.0)	<0.001
Capecitabine based	631 (21.6)	1,334 (23.6)	0.04
Chemotherapy discontinuation	668 (22.9)	1,582 (28.0)	<0.001

BMI, body mass index; CCI, Charlson's comorbidity index; ASA, American Society of Anesthesiologists; mean ± standard deviation

© 2025 Bong JW et al. *JAMA Network Open*.

eTable 2. Chemotherapy regimens according to pathological stage

Regimens	Stage II<br>N = 2,913 (%)	Stage III<br>N = 5,648 (%)	P-value
FL	977 (33.5)	360 (6.4)	<0.001
FOLFOX	659 (22.6)	2,048 (36.3)	<0.001
mFOLFOX	646 (22.2)	1,906 (33.7)	<0.001
Capecitabine	604 (20.7)	655 (11.6)	<0.001
CAPOX	27 (0.9)	679 (12.0)	<0.001

FL is administered as a 2-h infusion of leucovorin (200 mg/m²), followed by a bolus of fluorouracil (400 mg/m²) and then a 22-h continuous infusion of fluorouracil (600 mg/m²) on two consecutive days every 14 days, for a total of 12 cycles.

FOLFOX is administered as oxaliplatin (85 mg/m² IV over 2 h on day 1) simultaneously with leucovorin. Leucovorin (200 mg/m² IV) was followed by a fluorouracil bolus (400 mg/m² IV) and then a 22-h continuous infusion of fluorouracil (600 mg/m² IV) for 2 consecutive days, every 14 days, for a total of 12 cycles.

mFOLFOX is administered as oxaliplatin (85 mg/m² IV over 2 h on day 1), leucovorin (400 mg/m² IV over 2 h) and fluorouracil (400 mg/m² IV bolus, followed by a 46-h continuous infusion of 2,400 mg/m²) every 14 days for 12 cycles.

CAPOX is administered as oxaliplatin (130 mg/m²) intravenously on day 1 and capecitabine (1,000 mg/m²) orally twice daily for 14 days, followed by 7 days of rest (3-week cycles, total eight cycles).

Capecitabine is administered as oral capecitabine 1,000–1,250 mg/m² twice daily for 14 days, followed by 7 days of rest, in 3-week cycles for up to eight cycles.

© 2025 Bong JW et al. *JAMA Network Open*.

eTable 3. Clinical characteristics of patients with stage III colon cancer aged >70 years according to oxaliplatin use before and after adjustment using propensity score matching

Before propensity score matching					After propensity score matching				
Characteristics	Non-oxaliplatin, N = 662	Oxaliplatin, N = 1,055	P-value	SMD	Characteristics	Non-oxaliplatin, N = 300	Oxaliplatin, N = 300	P-value	SMD
Age, years	77.4 ± 4.1	74.8 ± 3.3	<0.001	0.641	Age, years	76.1 ± 3.7	76.0 ± 4.1	0.80	-0.04
Sex, male	326 (49.2)	600 (56.9)	0.002	-0.076	Sex, male	156 (52.0)	153 (51.0)	0.87	0.005
BMI, kg/m^2			0.45	-0.027	BMI, kg/m^2			0.93	0.02
Normal	251 (37.9)	428 (40.6)			Normal	123 (41.0)	126 (42.0)		
Underweight	45 (6.8)	61 (5.8)			Underweight	21 (7.0)	19 (6.3)		
~~Obese~~Overweight	366 (55.3)	566 (53.6)			Overweight~~Obese~~	156 (52.0)	155 (51.7)		
Emergency operation	26 (3.9)	36 (3.4)	0.672	-0.005	Emergency operation	12 (4.0)	8 (2.7)	0.50	<0.001
CCI			0.03	-0.016	CCI			0.43	0.01
0	121 (18.3)	210 (19.9)			0	53 (17.7)	62 (20.7)		
1–2	295 (44.6)	503 (47.7)			1–2	136 (45.3)	145 (48.3)		
3–4	153 (23.1)	243 (23.0)			3–4	67 (22.3)	59 (19.7)		
≥5	93 (14.0)	99 (9.4)			≥5	44 (14.7)	34 (11.3)		
ASA			0.002	-0.004	ASA			0.46	0.02
1	45 (6.8)	76 (7.2)			1	21 (7.0)	23 (7.7)		
2	434 (65.6)	766 (72.6)			2	200 (66.7)	211 (70.3)		
≥3	183 (27.6)	213 (20.2)			≥3	79 (26.3)	66 (22.0)		
Tumor histology			0.32	0.013	Tumor histology			0.55	<0.001
Adenocarcinoma	642 (97.0)	1,009 (95.6)			Adenocarcinoma	289 (96.3)	290 (96.7)		
Mucinous adenocarcinoma	19 (2.9)	45 (4.3)			Mucinous adenocarcinoma	11 (3.7)	9 (3.0)		
Signet-ring cell carcinoma	1 (0.2)	1 (0.1)			Signet-ring cell carcinoma	0 (0.0)	1 (0.3)		
Number of harvested lymph nodes <12	21 (3.2)	37 (3.5)	0.81	-0.003	Number of harvested lymph nodes < 12	14 (4.7)	10 (3.3)	0.53	<0.001
Chemotherapy regimens					Chemotherapy regimens				
Capecitabine based	484 (73.1)	123 (11.7)	<0.001	0.615	Capecitabine based	125 (41.7)	119 (39.7)	0.68	0.008
Chemotherapy discontinuation	221 (33.4)	421 (39.9)	0.008	-0.0652	Chemotherapy discontinuation	103 (34.3)	104 (34.7)	1.000	0.04

BMI, body mass index; CCI, Charlson's commodity index; ASA, American Society of Anesthesiologist; SMD, standardized mean differences

© 2025 Bong JW et al. *JAMA Network Open.*

eTable 4. Clinical characteristics of patients with stage III colon cancer ≤70 years old according to oxaliplatin use before and after adjustment using propensity score matching

Before propensity score matching					After propensity score matching				
Variables	Non-oxaliplatin, N = 352	Oxaliplatin, N = 3,563	P-value	SMD	Variables	Non-oxaliplatin, N = 346	Oxaliplatin, N = 346	P-value	SMD
Age, years	60.9 ± 8.3	57.6 ± 8.8	<0.001	0.393	Age, years	60.8 ± 8.3	61.2 ± 7.1	0.54	0.07
Sex, male	233 (66.2)	2,119 (59.5)	0.016	0.067	Sex, male	227 (65.6)	225 (65.0)	0.94	-0.009
BMI, kg/m^2			0.001	0.006	BMI, kg/m^2			0.56	0.01
Normal	138 (39.2)	1,376 (38.6)			Normal	137 (39.6)	132 (38.2)		
Underweight	31 (8.8)	155 (4.4)			Underweight	27 (7.8)	21 (6.1)		
Overweight~~Obese~~	183 (52.0)	2,032 (57.0)			Overweight~~Obese~~	182 (52.6)	193 (55.8)		
Emergency operation	4 (1.1)	149 (4.2)	0.008	0.031	Emergency operation	4 (1.2)	4 (1.2)	1.00	<0.001
CCI			0.002	-0.072	CCI			0.89	0.02
0	102 (29.0)	1,289 (36.2)			0	101 (29.2)	97 (28.0)		
1−2	158 (44.9)	1,614 (45.3)			1−2	154 (44.5)	150 (43.4)		
3−4	64 (18.2)	451 (12.7)			3−4	63 (18.2)	66 (19.1)		
≥5	28 (8.0)	209 (5.9)			≥5	28 (8.1)	33 (9.5)		
ASA			0.074	-0.050	ASA			0.44	0.02
1	80 (22.7)	986 (27.7)			1	78 (22.5)	81 (23.4)		
2	233 (66.2)	2,266 (63.6)			2	229 (66.2)	236 (68.2)		
≥3	39 (11.1)	311 (8.7)			≥3	39 (11.3)	29 (8.4)		
Cell type			0.197	0.020	Cell type			1.00	<0.001
Adenocarcinoma	344 (97.7)	3,411 (95.7)			Adenocarcinoma	338 (97.7)	338 (97.7)		
Mucinous adenocarcinoma	7 (2.0)	131 (3.7)			Mucinous adenocarcinoma	7 (2.0)	7 (2.0)		
Signet-ring cell carcinoma	1 (0.3)	21 (0.6)			Signet-ring cell carcinoma	1 (0.3)	1 (0.3)		
Number of harvested lymph nodes <12	14 (4.0)	85 (2.4)	0.102	0.016	Number of harvested lymph nodes < 12	12 (3.5)	12 (3.5)	1.000	0.02
Chemotherapy regimens					Chemotherapy regimens				
Capecitabine based	170 (48.3)	555 (15.6)	<0.001	0.327	Capecitabine based	164 (47.4)	161 (46.5)	0.88	-0.01
Chemotherapy discontinuation	73 (20.7)	862 (24.2)	0.166	-0.0345	Chemotherapy discontinuation	72 (20.8)	58 (16.8)	0.21	0.03

BMI, body mass index; CCI, Charlson's comorbidity index; ASA, American Society of Anesthesiologist; SMD, standardized mean differences

© 2025 Bong JW et al. *JAMA Network Open*.

eTable 5. Sensitivity analyses on the risk of overall mortality in patients with stage III colon cancer after excluding events that occurred within specified periods after the propensity score matching

**Washout period**	**Non-oxaliplatin**	**Oxaliplatin**	**P value**
Age ≤70 years			
6 months	1.00 (reference)	0.60 (0.41–0.90)	0.01
1 year	1.00 (reference)	0.60 (0.40–0.91)	0.02
Age >70 years			
6 months	1.00 (reference)	0.90 (0.66–1.23)	0.51
1 year	1.00 (reference)	0.83 (0.60–1.15)	0.27

Data are adjusted hazard ratio evaluated using Cox proportional hazards regression after adjustments for age, sex, body mass index, emergency, Charlson's comorbidity index, American Society of Anesthesiologists classification, tumor histology, number of harvested lymph nodes, chemotherapy regimen, chemotherapy discontinuation.

© 2025 Bong JW et al. *JAMA Network Open*.

eTable 6. Stratified analyses of overall survival in patients with stage III colon cancer receiving non-oxaliplatin versus oxaliplatin-combined adjuvant chemotherapy after propensity score matching

Variable	Non-oxaliplatin	Oxaliplatin	P for interaction
Age ≤70 years			
Sex			0.17
Male	1.00 (reference)	0.56 (0.35–0.71)	
Female	1.00 (reference)	0.74 (0.34–1.56)	
Body mass index			0.58
<23 kg/m^2	1.00 (reference)	0.70 (0.40–1.2)	
≥23 kg/m^2	1.00 (reference)	0.53 (0.30–0.91)	
Emergency operation			0.62
Yes	1.00 (reference)	N/A	
No	1.00 (reference)	0.59 (0.40-0.88)	
Charlson's comorbidity index			0.10
≤2	1.00 (reference)	0.64 (0.42–0.98)	
>2	1.00 (reference)	0.46 (0.15–1.36)	
ASA classification			0.39
1	1.00 (reference)	0.86 (0.34–2.15)	
≥2	1.00 (reference)	0.58 (0.37–0.91)	
Histological type			0.23
Adenocarcinoma	1.00 (reference)	0.65 (0.44–0.97)	
Others	1.00 (reference)	N/A	
Number of harvested lymph nodes			0.72
<12	1.00 (reference)	N/A	
≥12	1.00 (reference)	0.61 (0.41–0.91)	
Chemotherapy regimen			0.30
Capecitabine based	1.00 (reference)	0.91 (0.54–1.55)	
Fluorouracil based	1.00 (reference)	0.59 (0.40–0.88)	
Chemotherapy discontinuation			0.67
Yes	1.00 (reference)	0.65 (0.41–1.02)	
No	1.00 (reference)	0.48 (0.22–0.93)	
Age>70 years			
Sex			0.97
Male	1.00 (reference)	0.92 (0.60–1.43)	
Female	1.00 (reference)	0.99 (0.63–1.55)	
Body mass index			0.26
<23 kg/m^2	1.00 (reference)	1.06 (0.70–1.60)	
≥23 kg/m^2	1.00 (reference)	0.69 (0.44–1.08)	

© 2025 Bong JW et al. *JAMA Network Open*.

Emergency operation			0.04
Yes	1.00 (reference)	N/A	
No	1.00 (reference)	0.96 (0.70–1.30)	
Charlson’s comorbidity index			0.58
≤2	1.00 (reference)	0.84 (0.61–1.16)	
>2	1.00 (reference)	0.87 (0.33–2.33)	
ASA classification			0.47
1	1.00 (reference)	2.86 (0.64–12.80)	
≥2	1.00 (reference)	0.84 (0.62–1.15)	
Histological type			0.12
Adenocarcinoma	1.00 (reference)	0.91 (0.67–1.24)	
Others	1.00 (reference)	N/A	
Number of harvested lymph nodes			0.76
<12	1.00 (reference)	0.16 (0.008–3.02)	
≥12	1.00 (reference)	0.88 (0.65–1.20)	
Chemotherapy regimen			0.79
Capecitabine based	1.00 (reference)	0.92 (0.60–1.41)	
Fluorouracil based	1.00 (reference)	0.86 (0.62–1.15)	
Chemotherapy discontinuation			0.33
Yes	1.00 (reference)	0.96 (0.65–1.42)	
No	1.00 (reference)	0.79 (0.48–1.29)	

Data are adjusted hazard ratio evaluated using Cox proportional hazards regression after adjustments for age, sex, body mass index, emergency, Charlson’s comorbidity index, American Society of Anesthesiologists classification, tumor histology, number of harvested lymph nodes, chemotherapy regimen, chemotherapy discontinuation; N/A means “not available.” ASA, American Society of Anesthesiologists; 5-FU, 5-fluorouracil

© 2025 Bong JW et al. *JAMA Network Open*.

eTable 7. Adjusted hazard ratio of chemotherapy discontinuation for overall survival according to age cut-off

Patients	aHR	95% CI	P-value
Age≤70 years	1.79	1.56, 2.06	<0.001
Age>70 years	1.54	1.33, 1.79	<0.001

aHR: adjusted hazard ratio, CI: confidence interval

Data are adjusted hazard ratio evaluated using Cox proportional hazards regression after adjustments for age, sex, body mass index, emergency, Charlson's comorbidity index, American Society of Anesthesiologists classification, tumor histology, number of harvested lymph nodes, chemotherapy regimen

© 2025 Bong JW et al. *JAMA Network Open*.

eTable 8. Oxaliplatin dose and adjusted hazard ratio for overall survival in patient with oxaliplatin discontinuation according to age cut-off

	Oxaliplatin dose	N (%)	aHR	95% CI	P-value
Age≤70 years N = 2,701	<1.00	124 (4.6)	1.19	0.81, 1.76	0.37
	<0.85	98 (3.6)	1.30	0.85, 1.97	0.22
	<0.80	78 (2.9)	1.72	1.12, 2.62	0.01
Age>70 years N = 634	<1.00	31 (4.9)	1.27	0.72, 2.25	0.39
	<0.85	23 (3.6)	1.10	0.55, 2.17	0.79
	<0.80	18 (2.8)	1.52	0.76, 3.04	0.24

Oxaliplatin dose: actual oxaliplatin cycles / planned oxaliplatin cycles

Oxaliplatin-discontinuation: oxaliplatin dose < 1 and chemotherapy discontinuation = 1 (oxaliplatin discontinuation without fluoropyrimidine discontinuation)

aHR: adjusted hazard ratio, CI: confidence interval

Data are adjusted hazard ratio evaluated using Cox proportional hazards regression after adjustments for age, sex, body mass index, emergency, Charlson's comorbidity index, American Society of Anesthesiologists classification, tumor histology, number of harvested lymph nodes

© 2025 Bong JW et al. *JAMA Network Open*.

eFigure. Discontinuation rates according to age in patients with stage III disease

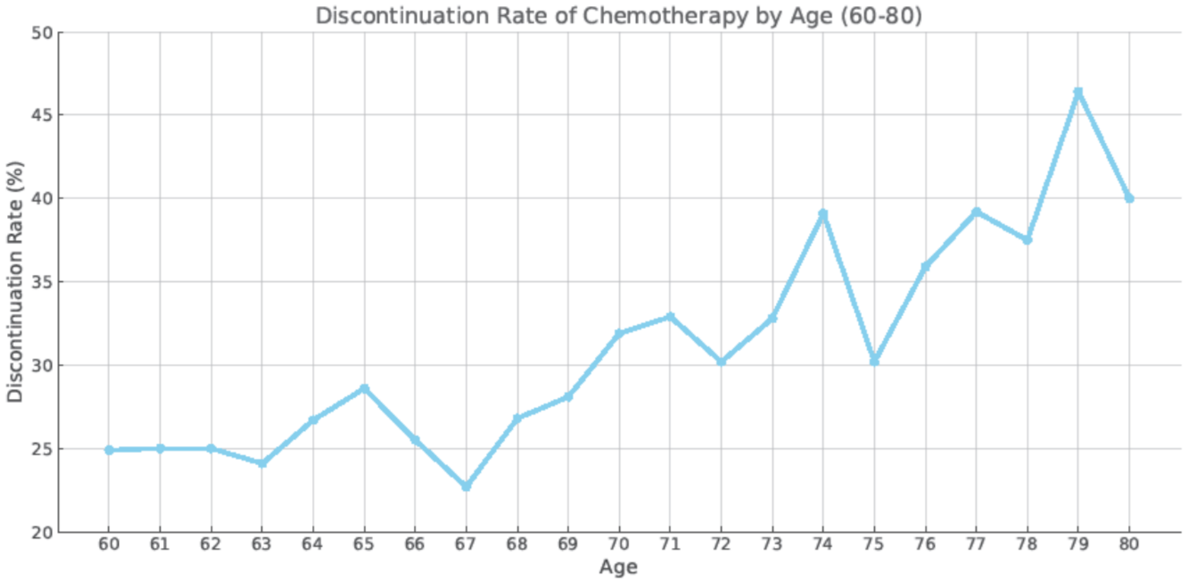

© 2025 Bong JW et al. *JAMA Network Open*.
