# Supplementary material for: Older Age Threshold for Oxaliplatin Benefit in Stage II to III Colorectal Cancer
Source: JAMA Netw Open. 2025 Aug 6;8(8):e2525660. doi: 10.1001/jamanetworkopen.2025.25660 (PMC12329608; doi:10.1001/jamanetworkopen.2025.25660)
Supplement: Supplement 2. — Data Sharing Statement [file jamanetwopen-e2525660-s002.pdf]

Data Sharing Statement

Bong. Older Age Threshold for Oxaliplatin Benefit in Stage II to III Colorectal Cancer. *JAMA Netw Open*. Published August 06, 2025. doi:10.1001/jamanetworkopen.2025.25660

Data
Data available: No

Additional Information
Explanation for why data not available: The data used in this study were obtained from HIRA and are accessible only to users with prior approval from HIRA.
